# A hypergraph neural network for prioritizing Alzheimer’s disease risk genes

**DOI:** 10.3389/fgene.2025.1668200

**Published:** 2025-09-19

**Authors:** Meng Ma, Chao Deng, Yan Liu, Qingqing Cao, Fang Liu, Yan Zhang

**Affiliations:** ^1^ College of Information Engineering, Hunan Open University, Changsha, China; ^2^ School of Computer Science and Engineering, Central South University, Changsha, China; ^3^ School of Computer Science and Engineering, Hunan University of Information Technology, Changsha, China

**Keywords:** Alzheimer’s disease, higher-order associations, hypergraph neural network, hypergraph, disease gene

## Abstract

Identifying the complex genetic architecture of Alzheimer’s disease (AD) is critical for understanding its pathophysiology. While network-based computational methods assist in this task, they primarily model simple pairwise gene interactions and fail to capture the higher-order associations of genes that drive complex diseases. To address this limitation, we introduce HyperAD, a novel hypergraph neural network framework designed to predict AD risk genes by explicitly modeling these higher-order associations of genes. HyperAD constructs a hypergraph in which functional gene sets from databases such as MSigDB form hyperedges, and uses a two-stage hypergraph message passing neural network to extract high-order association information from the hypergraph. Comprehensive evaluations demonstrate that HyperAD significantly outperforms state-of-the-art methods. We validate the prediction results of HyperAD through multiple lines of evidence. HyperAD-predicted genes are enriched in AD-related biological processes and have significant associations with known related genes in terms of sequence similarity, protein interaction, and miRNA. In addition, their protein expression levels are significantly altered in the brains of AD patients, and they contain both known risk sites and new, high-confidence candidate genes. HyperAD provides a more accurate and biologically insightful tool for prioritizing genes and unraveling the complex genetic landscape of AD.

## 1 Introduction

Alzheimer’s disease (AD) is a progressive and devastating neurodegenerative disorder that represents the most common cause of dementia worldwide. As global populations age, the societal and economic burden of AD is escalating, making the search for effective therapeutic strategies a global health priority. The disease has a strong genetic component, with heritability estimates ranging from 60% to 80% (Bellenguez et al., 2022). While the discovery of rare, highly penetrant mutations in genes such as APP, PSEN1, and PSEN2 confirmed the role of genetics in early-onset familial AD, the genetic architecture of the more common late-onset AD (LOAD) is far more complex and remains incompletely understood (Spina et al., 2021). Therefore, the systematic identification and prioritization of novel AD risk genes are fundamental to unraveling the intricate pathophysiology of the disease, identifying new biological pathways for investigation, and ultimately developing targeted therapies.

Genome-wide association studies (GWAS) have been the primary engine for discovering common genetic variants associated with AD (Bellenguez et al., 2022; Dalmasso et al., 2024). To date, these efforts have successfully identified dozens of risk loci. For instance, a landmark GWAS meta-analysis by (Jansen et al., 2019) identified 29 risk loci, implicating potential pathogenic genes such as ADAMTS4 and KAT8. These findings highlighted the enrichment of risk genes in immune-related cell types, particularly microglia, and their involvement in pathways such as lipid processing and amyloid-beta precursor protein degradation. More recently, a large two-stage GWAS by (Bellenguez et al., 2022) further expanded the genetic map of AD, identifying 75 risk loci, 42 of which were novel, including those near SORT1 and ANK3. Despite these successes, GWAS have inherent limitations. The stringent p-value thresholds required to correct for multiple testing can lead to high false-negative rates, while the analysis often identifies large genomic loci containing numerous genes, making it challenging to pinpoint the true causal gene (McCarthy et al., 2008; Lee et al., 2011). Furthermore, these studies demand massive, well-phenotyped patient cohorts, which are expensive and time-consuming to assemble. Consequently, computational approaches are essential to complement GWAS findings, helping to prioritize the most promising candidates from thousands of potential genes for functional validation.

In recent years, a variety of computational methods have been developed to predict disease risk genes. The majority of these methods operate on the “guilt-by-association” principle, which posits that genes involved in the same disease are likely to be functionally related and thus exhibit proximity or strong connections within biological networks (Cowen et al., 2017; Yao et al., 2018; Valdeolivas et al., 2019; Ata et al., 2021). Methods based on protein-protein interaction (PPI) networks or functional gene networks have been applied to this problem. In the study of disease gene identification, network-based machine learning approaches have proven effective. For instance (Yao et al., 2018), developed a Bayesian framework to construct tissue-specific functional gene networks by integrating multiple types of gene association data. These networks were then used as features to train machine learning models for identifying disease-associated genes. Similarly (Krishnan et al., 2016), proposed a machine learning approach that leverages a brain-specific FGN to prioritize candidate genes for autism spectrum disorder. In another approach (Tran et al., 2020), introduced DiGI, a node kernel-based method that generates gene features by integrating information from multiple heterogeneous networks, which are subsequently used by a Support Vector Machine (SVM) for prediction. More recently (Mancuso et al., 2022), developed GenePlexus, a framework that utilizes biological networks and a semi-supervised learning approach to identify novel disease-gene associations. These models typically leverage pairwise, or binary, associations between genes to propagate risk information across the network, identifying novel candidates based on their connectivity to known disease genes.

However, a significant limitation of these biological network-based approaches is their reliance on pairwise interactions. The pathophysiology of a complex polygenic disease like AD is not driven by a series of independent gene-gene interactions, but rather by the intricate and collective interplay of multiple genes within complex biological pathways and functional modules (Sims et al., 2020). By modeling only binary relationships, existing methods may fail to capture the higher-order associative patterns that characterize these multi-gene functional units (Li et al., 2022; Deng et al., 2024). This oversight means that crucial information embedded within functionally cohesive gene sets—where the coordinated action of several genes is necessary for a biological outcome—is largely ignored.

To address this critical gap, we introduce HyperAD, a novel hypergraph neural network framework for predicting AD risk genes by explicitly modeling higher-order gene relationships. We leverage the comprehensive gene set collections from the Molecular Signatures Database (MSigDB) (Liberzon et al., 2015), which curates functionally related gene groups based on diverse biological evidence, including chromosomal position, Gene Ontology (GO) annotations (Consortium, 2004), KEGG pathways (Kanehisa and Goto, 2000), and Human Phenotype Ontology (HPO) associations (Köhler et al., 2021). We structure this information using a hypergraph, a powerful data structure where a single hyperedge can connect any number of nodes (Agarwal et al., 2006; Zhou et al., 2006). This representation provides a natural and superior way to model the complex, many-to-many relationships inherent in biological systems compared to traditional graphs. To exploit this rich representation, we designed a two-stage message-passing hypergraph neural network that effectively learns and aggregates features from these complex, higher-order structures. By capturing the collective context of gene function, HyperAD is designed to more accurately prioritize novel AD risk genes and provide deeper insights into the multi-gene mechanisms underlying the disease.

To assess the effectiveness and robustness of our proposed framework, we conduct a series of comprehensive experiments. HyperAD is compared with a series of state-of-the-art AD risk gene prediction methods, and the results demonstrate its superior predictive performance. In addition to these comparative analyses, we also conducted several validation studies to confirm the biological relevance and accuracy of the HyperAD-predicted AD risk gene. Functional enrichment analyses revealed that the HyperAD-predicted AD risk genes are significantly enriched in AD-associated biological processes and share a substantial number of network connections with known AD genes, underscoring their strong functional relevance to the disease. Furthermore, we integrate proteomic expression data into our validation pipeline, which shows that the HyperAD-predicted AD risk genes correspond to proteins that are significantly differentially expressed in AD brain tissues.

## 2 Materials and methods

### 2.1 Datasets

To construct the hypergraph, we obtain gene sets from the Molecular Signatures Database (v7.4), one of the most comprehensive and widely used collections of annotated gene sets (Liberzon et al., 2015). MSigDB aggregates data from numerous sources to cover diverse biological contexts, including chromosomal location, biological function, metabolic pathways, and regulatory targets (Subramanian et al., 2005; Liberzon et al., 2011). The database organizes gene sets into nine major collections (H and C1-C8) according to their origin and type. For this study, we use several of these key collections. These included Hallmark gene sets (H), which represent well-defined biological processes, and positional gene sets (C1), which group genes by chromosomal location. We also incorporate curated gene sets (C2) from pathway databases and the literature, alongside regulatory target gene sets (C3) that comprise genes sharing predicted targets for microRNAs or transcription factors. Additional information is derived from ontology gene sets (C5), which group genes based on GO and HPO terms, and computational gene sets (C4) derived from mining cancer-oriented microarray data. Finally, we include several signature-based collections: oncogenic gene sets (C6) from cancer gene perturbation studies, immunologic gene sets (C7) from immune system perturbations, and cell type gene sets (C8) derived from cluster markers in single-cell sequencing studies. The number and description of gene sets in each category are in Table 1.

**TABLE 1:** Brief introduction of nine major collections in MSigDB.

Collection	Description	Number of gene sets
H	Hallmark gene sets	50
C1	Positional gene sets	278
C2	Curated gene sets	6290
C3	Regulatory target gene sets	3731
C4	Computational gene sets	858
C5	Ontology gene sets	14,998
C6	Oncogenic signature gene sets	189
C7	Immunologic signature gene sets	5,219
C8	Cell type signature gene sets	671

To train our model and evaluate its performance, we establish a ground-truth dataset of genes associated with AD. The positive set is composed of 147 high-confidence AD risk genes, manually curated from several authoritative databases such as OMIM (Amberger et al., 2019), GWAS Catalog (Sollis et al., 2023), DisGeNet (Piñero et al., 2020), and AlzGene (Bertram et al., 2007). Constructing a reliable negative set—genes with no association to AD is inherently challenging. To address this, we adopt a stringent filtering strategy. First, we compile a comprehensive list of potential AD-associated genes from the databases mentioned above. We then create a negative (non-AD) gene pool by removing all these potential AD genes from the complete human genome. To mitigate class imbalance during model training, we randomly sampled 1,000 genes from this pool to form our final negative set.

### 2.2 Method

#### 2.2.1 Brief introduction of hypergraph

A weighted hypergraph is defined as G(V,E,W) , where $V=\left\{v_{1},v_{2},v_{3},\ldots ,v_{N}\right\}$ represents the set of N nodes in the hypergraph, $E=\left\{e_{1},e_{2},e_{3},\ldots ,e_{M}\right\}$ represents the set of M hyperedges in the hypergraph, and $diag\left(W\right)=\left[w\left(e_{1}\right),w\left(e_{2}\right),\ldots ,w\left(e_{m}\right)\right]$ is the diagonal matrix representing the weight of the hyperedge. W(e) is used to express the importance of the hyperedge e in the hypergraph. For an unweighted hypergraph, W can be regarded as the identity matrix. An edge in the graph can only represent the relationship between two nodes, but the hyperedges in the hypergraph can connect more than two nodes and can represent the relationship among multiple nodes. In order to describe the relationship between the nodes and the hyperedges, the hypergraph is usually represented by an incidence matrix H of N×M , and $H\left(v_{a},e_{b}\right)\in \left\{0,1\right\}$ indicates that whether the a -th node $v_{a}$ is belong to the b -th hyperedge $e_{b}$ :

$$H\left(v_{a},e_{b}\right)=\left\{\begin{array}{cc} 1\; & \;\;\text{if }v_{a}\in e_{b}\\ 0\; & \;\;\text{if }v_{a}\notin e_{b} \end{array}\right.$$ (1)

For a node $v_{a}\in V$ , the degree of the node is defined as:

$$d\left(v_{a}\right)=\underset{e\in E}{\sum }w\left(e\right)H\left(v_{a},e\right)$$ (2)

For an hyperedge $e_{b}\in E$ , the degree of the hyperedge is defined as:

$$\delta \left(e_{b}\right)=\underset{v\in V}{\sum }H\left(v,e_{b}\right)$$ (3)

And, we can define the $D_{v}\in R^{N\times N}$ and $D_{e}\in R^{M\times M}$ denote the diagonal matrices of the node degrees and the hyperedge degrees, respectively.

Kipf et al. (Kipf and Welling, 2017) propose the graph convolution network (GCN) through the local first-order approximation of the spectral graph convolution. A GCN layer is define as:

$$X^{\left(l+1\right)}=\sigma \left({\tilde{D}}^{-\frac{1}{2}}\tilde{A}{\tilde{D}}^{-\frac{1}{2}}X^{\left(l\right)}\Theta^{\left(l\right)}\right)$$ (4)

where $\tilde{A}$ denotes the adjacency matrix with self connections and $\tilde{D}$ denotes the degree matrix of the graph. $\Theta^{\left(l\right)}$ is a learnable weight matrix and σ denotes the activation function.

The Laplace matrix is crucial in the spectral method of graphs. For an graph, the Laplace matrix is defined as Δ=D−A . Zhou et al. (2006) use the Laplacian matrix of graphs for reference, introduce the concept of the Laplacian matrix in the hypergraph, and define it as:

$$\Delta =I-D_{v}^{-\frac{1}{2}}HWD_{e}^{-1}H^{\text{T}}D_{v}^{-\frac{1}{2}}$$ (5)

Feng et al. (2019) bring the hypergraph Laplacian matrix Δ into the traditional spectrogram convolution, and define the spectrogram convolution operation of the hypergraph $g*x=\Phi g\left(\Lambda \right)\Phi^{\text{T}}x$ . At the same time, Feng et al. (2019) use the second-order Chebyshev inequality to approximate g∗x , and the HGNN is proposed. Finally, a hypergraph convolution layer is defined as:

$$X^{\left(l+1\right)}=\sigma \left(D_{v}^{-\frac{1}{2}}HWD_{e}^{-1}H^{\text{T}}D_{v}^{-\frac{1}{2}}X^{\left(l\right)}\Theta^{\left(l\right)}\right)$$ (6)

where $D_{v}$ denotes the node degree matrix and $D_{e}$ denotes the hyperedge degree matrix. $\Theta^{\left(l\right)}$ is a learnable weight matrix and σ is the activation function.

#### 2.2.2 Framework of HyperAD

**FIGURE 1:**
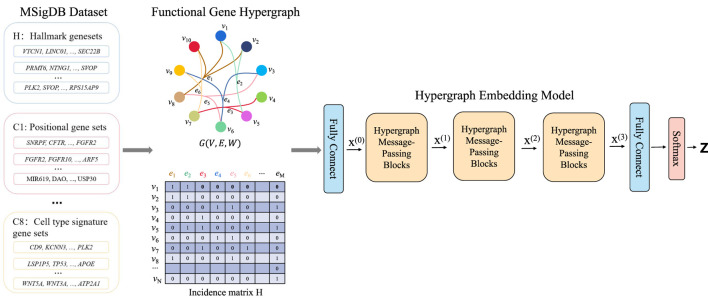
The HyperAD framework for AD gene prioritization. The framework begins by constructing a hypergraph from diverse gene sets collected from the MSigDB, where each gene set forms a hyperedge connecting its member genes. This hypergraph is then fed into a deep learning model composed of multiple hypergraph message-passing modules. These modules iteratively aggregate information across the hyperedges, allowing the model to learn complex associative patterns that are often missed by traditional pairwise network models. Finally, the model predicts a genome-wide AD risk score for each gene, enabling the prioritization of novel candidates.

HyperAD is a novel, end-to-end deep learning framework designed to identify genes associated with AD by modeling higher-order assocation among genes (Figure 1). It uniquely frames gene prioritization as a learning task on a hypergraph, where genes are represented as nodes and diverse gene sets from the MSigDB database as hyperedges. The core of HyperAD is a novel two-stage hypergraph message-passing module that learns gene representations by explicitly modeling the flow of information from genes to pathways and back. Moreover, we propose an AD-specific weighting mechanism that dynamically focuses the model on the most relevant biological contexts during learning, leading to highly specific and robust gene embeddings for AD-associated gene prediction. Finally, the learned gene representations are used for AD gene prioritization.

First, we use a hypergraph to structurally represent the gene sets from the MSigDB database. This database contains gene sets from various sources, including GO terms, KEGG pathways, and HPO. Each gene set consists of genes that are associated in some way. For example, a KEGG pathway indicates that multiple genes are involved in a common signaling pathway. In bioinformatics and computational biology, graphs or network structures are commonly used to describe the relationships between proteins, genes, or other biological entities. However, for annotated gene sets, standard graph structures are not suitable because they cannot represent the higher-order relationships among multiple genes within a single gene set. To accurately describe the complex associations of genes in these sets and to fully utilize their higher-order relationships, our method, HyperAD, uses a hypergraph structure to represent and integrate different types of gene sets. We define the hypergraph constructed using gene sets as G(V,E,W) , the node $V=\left\{v_{1},v_{2},v_{3},\ldots ,v_{N}\right\}$ in the hypergraph represents N genes, and the hyperedge $E=\left\{e_{1},e_{2},e_{3},\ldots ,e_{M}\right\}$ represents M annotated gene sets. For the incidence matrix $H\in R^{N\times M}$ of the hypergraph, we define that if the a -th gene $v_{a}$ belongs to the b -th annotated gene set $e_{b}$ , then $H\left(v_{a},e_{b}\right)=1$ , otherwise it is 0. The incidence matrix H of the hypergraph G is constructed from the inclusion relationship between genes and annotated gene sets.

Next, we detail the architecture of our model, HyperAD. The model first projects the initial one-hot encoded gene features $x\in R^{N\times N}$ into a dense, low-dimensional embedding space $x_{v}^{\left(0\right)}\in R^{N\times d}$ using a non-linear transformation layer:

$$x_{v}^{\left(0\right)}=ReLU\left(x\Theta_{0}+b_{0}\right)$$ (7)

where $\Theta_{0}$ represents a learnable parameter matrix, and $b_{0}$ represents the bias value of the layer. The x is the initial feature vector for gene v , and $x_{v}^{\left(0\right)}$ is its embedding after the initial transformation.

The resulting gene embeddings are then processed by a series of our core two-stage hypergraph message-passing blocks. Within each block, a node-centric perspective reveals how higher-order associations are learned by explicitly modeling the information flow from genes to gene sets and back. The update rules for a single node v and hyperedge e (from layer l to l+1 ) are defined as follows:

Node-to-Hyperedge Aggregation. In this stage, each hyperedge e updates its feature vector $y_{e}$ by aggregating messages from all its member nodes v . This message is the normalized feature vector of the node:

$$y_{e}^{\left(l\right)}=\sigma \left(\underset{v\in N\left(e\right)}{\sum }\left(\frac{x_{v}^{\left(l\right)}}{d\left(v\right)}\right)\Theta_{v\to e}^{\left(l\right)}\right)$$ (8)

Where $x_{v}^{\left(l\right)}$ is the feature vector of node v at layer l and $y_{e}^{\left(l\right)}$ is the feature vector of hyperedge e . The N(e) is the set of all nodes contained within the hyperedge e . The d(v) is the degree of node v , used for normalization. The $\Theta_{v\to e}^{\left(l\right)}$ is the learnable parameter matrix. The σ denotes a non-linear activation function, such as ReLU .

Hyperedge-to-Node Aggregation. Subsequently, each node v updates its feature vector $x_{v}$ by aggregating messages from all the hyperedges e it belongs to. In a hypergraph, the weight of a hyperedge signifies its relevance to the classification task. An unweighted hypergraph, such as the one we initially construct, implicitly assumes that all hyperedges—representing diverse biological pathways—contribute equally to predicting AD-associated genes. This assumption is biologically implausible. The etiology of complex diseases like Alzheimer’s is known to be driven by a specific subset of biological processes and molecular functions. For instance, pathways related to synaptic function or neuro-inflammation are critically relevant to AD, whereas some others may not be involved. Treating these distinct pathways with uniform importance would introduce significant noise and overlook crucial disease-specific signals. Therefore, to align our model with this biological reality and enhance its predictive power, we propose a weighted aggregation of hyperedge information. This approach assigns greater importance to pathways more significantly associated with AD. The mechanism is formalized as follows.

$$w\left(e\right)=\frac{\sum_{v\in V}H\left(v,e\right)f\left(v,V_{d}\right)}{\sum_{v\in V}H\left(v,e\right)}$$ (9)

$$f\left(v,V_{d}\right)=\left\{\begin{array}{cc} 1\; & \;\;\text{if }v\in V_{d}\\ 0\; & \;\;\text{if }v\notin V_{d} \end{array}\right.$$ (10)

where w(e) denotes the weight of the hyperedge e , and $V_{d}$ denotes the known disease-associated gene. The $f\left(v,V_{d}\right)$ is used to indicate whether gene v belongs to $V_{d}$ . This step is followed by a residual connection to preserve the node’s original information (He et al., 2016). Therefore, the specific formula of the message passing mechanism from hyperedge to node in HyperAD is as follows:

$$x_{v}^{\left(l+1\right)}=\sigma \left(\underset{e\in E\left(v\right)}{\sum }\left(w\left(e\right)\cdot y_{e}^{\left(l\right)}\right)\Theta_{e\to v}^{\left(l\right)}+x_{v}^{\left(l\right)}\right)$$ (11)

Where $x_{v}^{\left(l+1\right)}$ is the feature vector of node v at layer l+1 . The E(v) is the set of all hyperedges that contain node v . The w(e) is the pre-computed weight of hyperedge e , modulating the message importance. The term $+x_{v}^{\left(l\right)}$ represents the residual connection. The σ denotes a non-linear activation function.

Finally, HyperAD inputs the gene feature matrix $x_{v}^{\left(l\right)}\in R^{N\times d}$ to the fully connected layer and uses the softmax layer to get the prediction result of the model, the formula is as follows:

$$Z=Softmax\left(x_{v}^{\left(l\right)}\Theta_{1}+b_{1}\right)$$ (12)

where $Z\in R^{N\times 2}$ represents the predicted risk score of each gene, and we can use it to prioritize the degree of association between AD and genes. We use cross-entropy as the loss function of the model and use the Adam algorithm to optimize all parameters during the training.

## 3 Results

### 3.1 Benchmarking of HyperAD

To demonstrate the superiority of HyperAD, we conduct a comprehensive benchmark against six state-of-the-art methods for disease gene prediction: FGN, RWRM, DiGI, GCN-GENE, GSI, and GenePlexus. FGN (Yao et al., 2018) first constructs a tissue-specific functional gene network by integrating various evidence sources, such as coexpression and protein-protein interactions, within a Bayesian framework; this network is then used to train an SVM classifier. c DiGI, a node kernel-based approach, generates powerful gene features by applying a graph kernel to multiple heterogeneous networks and then integrates these features to train an SVM for prediction (Tran et al., 2020). GCN-GENE (Zhang et al., 2022) represents a deep learning approach, utilizing a Graph Convolutional Network to learn node embeddings by convolving over a biological network structure while using gene expression data as initial node features. GSI (Li et al., 2022) first constructs a gene signal matrix where each row is a gene and each column represents a biological signal derived from gene-set membership, which is then fed into a machine learning model; for our experiments, we use its high-performing Random Forest-based implementation. Lastly, GenePlexus (Mancuso et al., 2022) employs a semi-supervised learning strategy on a large-scale human gene interaction network, propagating labels from known disease genes to predict novel associations. For a fair and robust evaluation, all methods are trained and tested on identical positive and negative gene sets under a rigorous ten-repetition, five-fold cross-validation scheme. We evaluate performance using the Area Under the Receiver Operating Characteristic Curve (AUROC) and the Area Under the Precision-Recall Curve (AUPRC), reporting the mean and standard deviation across all 50 folds to ensure a reliable comparison.

**FIGURE 2:**
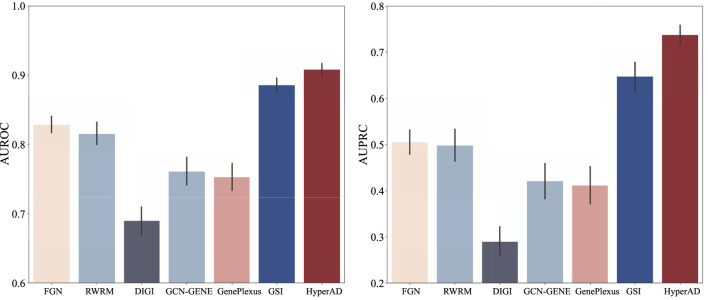
Benchmarking experimental results of HyperAD and six advanced AD gene prediction methods.

As illustrated in Figure 2, HyperAD demonstrates superior performance, significantly outperforming all state-of-the-art methods for AD-associated gene prediction. Compared to approaches that rely on traditional pairwise biological networks such as FGN, DiGI, RWRM, GCN-GENE, and GenePlexus, HyperAD achieves substantial performance gains, with an increase of 7.9%–21.7% in AUROC and a remarkable 23.2%–44.7% in AUPRC. This result strongly suggests that higher-order associations captured within annotated gene sets provide a more accurate and informative representation of complex gene functions than pairwise interactions in conventional networks. Furthermore, HyperAD also significantly outperforms GSI, the most comparable baseline, improving AUROC by 2.3% and AUPRC by 9.0%. This comparison is particularly insightful because while both methods use the same raw gene-set membership information, their modeling strategies differ fundamentally. GSI treats the gene-set membership (the incidence matrix H) as a static feature matrix for a standard classifier, whereas HyperAD employs its two-stage message-passing architecture to model the hypergraph structure. This allows HyperAD to deeply explore higher-order neighborhood information and the local topology around each gene while simultaneously integrating disease-specific functional relevance through its weighted aggregation mechanism. The superior performance of HyperAD over GSI unequivocally demonstrates the power of this dynamic modeling approach. It underscores the critical importance of explicitly learning from the neighborhood context within the hypergraph. We also analyze the hyperparameter sensitivity of HyperAD (Supplementary Figure). Collectively, these results establish HyperAD as a highly effective framework and suggest that leveraging hypergraph neural networks to model higher-order biological relationships is a promising new direction for disease gene prediction.

### 3.2 Ablation studies of HyperAD

**FIGURE 3:**
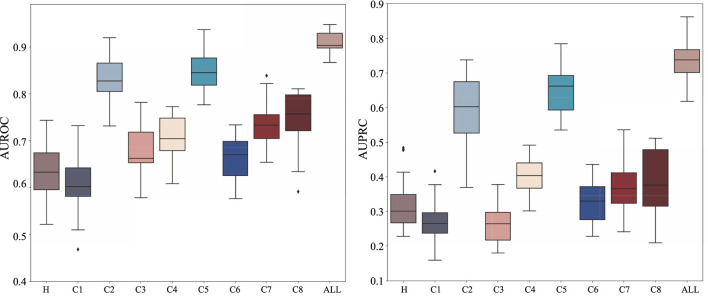
Ablation analysis results on the MSigDB database. We analyze and show the performance of HyperAD on different types of gene sets in the MSigDB database.

To understand the relative contribution of different types of biological information to HyperAD’s predictive performance, we conduct a comprehensive ablation study. In this analysis, we train and evaluate our model separately on each of the nine individual MSigDB collections (H and C1-C8) and compare their performance against the final integrated model that utilizes all collections simultaneously. As shown in Figure 3, the results demonstrate that the integrated model achieves the highest predictive accuracy, significantly outperforming any model trained on a single data source. This finding suggests that our model’s superior performance stems from its ability to synthesize diverse and complementary biological knowledge, from curated pathways to cell-type-specific signatures.

Upon closer checking of the models trained on individual collections, we find that the prediction performance is relatively better for curated (C2) and ontology (C5) gene sets. This indicates that the higher-order functional relationships derived from canonical pathway databases (C2) and formal biological ontologies like GO and HPO (C5) form the primary informational backbone for HyperAD’s ability to identify AD-associated genes. While other collections perform more modestly in isolation, their inclusion is not redundant. Instead, they act as a crucial source of Supplementary Material. For example, gene sets like positional (C1) and regulatory target (C3) likely provide essential context on genomic proximity and co-regulation that refines the core functional predictions. Therefore, we conclude that HyperAD’s strength lies in its hierarchical use of information: it leverages the rich, explicit functional knowledge in C2 and C5 as its foundation, while integrating contextual cues from the other collections to enhance its final predictions.

Furthermore, we conduct a second ablation study to specifically evaluate the contribution of our core AD-specific weighting module. In this experiment, we remove this module from the architecture, thereby treating all biological information with equal importance without disease-specific prioritization. As detailed in Table 2, the full HyperAD model demonstrates a significant performance gain compared to the ablated version, with an increase of 3.3% in AUROC and 12.3% in AUPRC. This result strongly highlights the critical role of the AD-specific weighting module in focusing the model on the most disease-relevant biological contexts, which substantially enhances its predictive accuracy.

**TABLE 2:** Ablation study result of AD-specific weighting module.

Model Configuration	AUROC	AUPRC
HyperAD w/o Weighting	0.881	0.653
HyperAD	0.914	0.766

### 3.3 HyperAD-predicted genes significantly enriched in AD-associated biological process

A key criterion for a successful disease gene prioritization model is its ability to rank genes involved in the core biological processes of the disease highly (Krishnan et al., 2016). To evaluate our model against this standard, we conduct a functional enrichment analysis. We use a set of 41 highly relevant GO biological processes (Di Paolo and Kim, 2011), which represent key AD pathways derived from enrichment analysis of known AD genes and filtered by AD risk factors and phenotypes. We then employ a decile enrichment test to systematically assess whether our ranked list of genes (after excluding known positives) is enriched for these AD-associated functions at its top tier.

**FIGURE 4:**
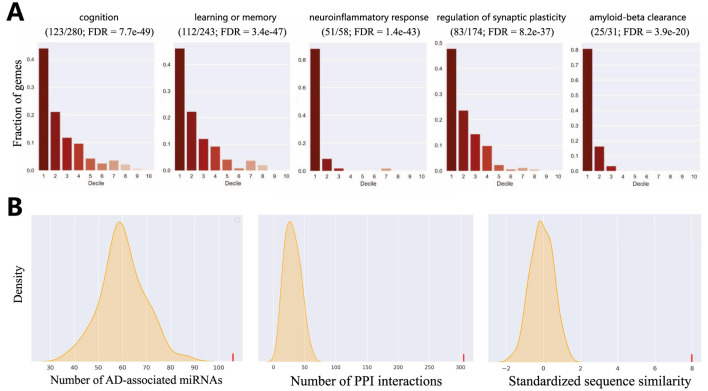
Evaluation of HyperAD prediction. **(A)** Evaluation of the overlap of AD-associated biological processes within the first decile of our predictions. Fractions of genes (y-axis) indicate the distribution of genes in the gene set in each decile of the predictions. **(B)** Statistical validation of our predictions using multiple biological networks.

The results from this analysis provide compelling evidence for our model’s biological validity. As shown in Figure 4A and detailed in Supplementary Table, we observed an overwhelming enrichment of these 41 core AD biological processes specifically within the first decile of our ranked list (i.e., the top 10% of scored genes). The significance of this finding lies not only in its statistical power but also in the nature of the enriched functions. We note that processes directly corresponding to hallmark AD pathologies were among the most significant, with exceptionally low FDR values indicating high confidence. These can be broadly categorized into two groups: those reflecting neuronal dysfunction, such as “cognition” (GO:0050890, FDR $=7.7\times 10^{-49}$ ), “learning or memory” (GO:0007611, FDR $=3.4\times 10^{-47}$ ), and “regulation of synaptic plasticity” (GO:0048167, FDR $=8.2\times 10^{-37}$ ); and those reflecting core molecular pathology, such as “amyloid-beta clearance” (GO:0097242, FDR $=3.9\times 10^{-20}$ ). This ability to recapitulate functions related to both macro-level phenotypes and micro-level molecular mechanisms of AD among the top-ranked genes clearly demonstrates that our model does not just find AD-associated genes, but precisely identifies those central to the disease’s functional network. This result substantially increases our confidence in prioritizing these top-ranked candidates for future experimental validation and drug target discovery.

### 3.4 HyperAD-predicted genes are significantly linked to known AD-associated genes

We further assess the AD-relevance of our top-ranked candidate genes using a network biology approach. We select the top 50 novel genes (after excluding known positive genes) and test whether they are significantly associated with the set of established AD genes in three distinct biological networks. These networks represent different biological dimensions: a protein sequence similarity network, the protein-protein interaction (PPI) network from the STRING database (Szklarczyk et al., 2015), and the miRNA-target interaction network from mirTarBase (Hsu et al., 2011). As shown in Figure 4B, the statistical analysis yields a highly significant result, indicating that the top 50 genes are strongly associated with known AD genes in all three networks (P-value <0.0001 ).

This highly consistent result carries significant biological implications. It suggests that our identified candidate genes have multiple functional links to known AD genes: they not only exhibit high sequence similarity, implying potential functional homology, but their protein products also tend to participate in common cellular interaction networks and pathways. Furthermore, they are likely subject to shared miRNA co-regulation at the post-transcriptional level. Therefore, this convergent evidence from diverse molecular layers strongly supports the conclusion that our predicted genes are functionally coupled with the pathobiology of AD, marking them as promising targets for future investigation.

### 3.5 Protein expression of HyperAD-predicted genes is associated with cognitive function

To bridge our computational predictions with real-world clinical pathology, we conduct a critical validation to assess whether the protein expression levels of our top-ranked candidate genes are directly associated with the progression of cognitive decline in AD. For this purpose, we leverage longitudinal proteomics data from the Religious Orders Study and Memory and Aging Project (ROSMAP) (Canchi et al., 2019), a deeply phenotyped and highly valuable cohort study (Synapse accession doi:10.7303/syn3219045). Based on the clinical diagnosis variable ‘dcfdx_lv’, we stratified the samples into three distinct cognitive groups: No Cognitive Impairment (NCI, n = 174), Mild Cognitive Impairment (MCI, n = 100), and AD (n = 104). Our central hypothesis is that if a gene is a key driver of AD, its encoded protein expression should exhibit a monotonic increase or decrease across the disease progression continuum from NCI to MCI to AD. To test the significance of such trends, we employ the Kendall’s Tau-b test, a non-parametric statistical method ideally suited for assessing monotonic relationships between ordinal variables.

**FIGURE 5:**
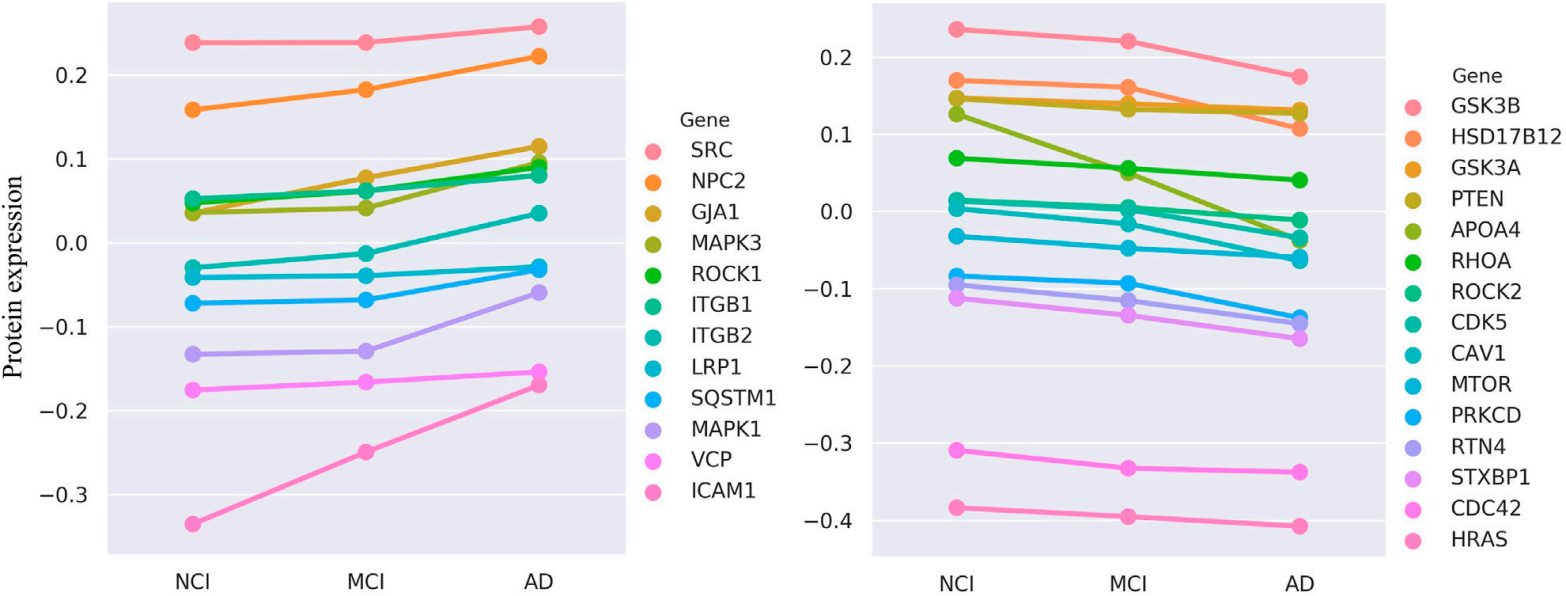
The evaluation results of ROSMAP data. Protein expression of 27 genes is monotonically increasing (n = 12) or decreasing (n = 15) in three stages.

Among our top 200 candidate genes (after excluding known positives), protein expression data are available for 67 in the ROSMAP dataset. The results are highly encouraging: of these 67 genes, we identify that a substantial proportion—27 genes (40.3%)—exhibit protein expression levels that are significantly and monotonically correlated with the severity of cognitive decline (Kendall’s Tau-b test, FDR <0.05 ). Of these, 12 show a positive correlation (expression increasing as cognitive function declines), while the remaining 15 show a negative correlation (Figure 5). This high percentage of significantly associated genes strongly suggests that the candidates identified by our model are not random but are coupled at the molecular level to the clinical progression of AD.

Deeper case studies further illuminate the biological significance of our model’s predictions. For instance, we find that the protein expression of CDK5 and GSK3B, two kinases known to play pivotal roles in AD, is significantly and negatively correlated with cognitive function. This aligns perfectly with their established biological roles, as the aberrant activation of these kinases is a central event driving the hyperphosphorylation of the microtubule-associated protein Tau (MAPT), leading to neurofibrillary tangles (Jayapalan and Natarajan, 2013). The ability of our model to independently identify these key regulators and confirm that their expression trend matches disease progression significantly bolsters its credibility. Furthermore, a particularly compelling finding involves APOA4. We not only find its expression to be negatively correlated with cognitive function but also note its colocalization on chromosome 11 with APOA1 and APOC3, which also appeared in our top-200 list. The decreased expression of this APOA1-APOC3-APOA4 gene cluster is known to be associated with AD risk (Lin et al., 2015). This result powerfully suggests that our HyperAD model can effectively utilize higher-order association information among multiple genes to identify functionally synergistic modules, a feat that is often beyond the reach of traditional single-gene or pairwise association methods.

## 4 Discussion

In this study, we introduced HyperAD, a hypergraph neural network framework designed to overcome a key limitation of existing methods for AD gene prioritization: their reliance on simple pairwise gene interactions. By explicitly modeling higher-order relationships derived from functional gene sets, HyperAD more accurately reflects the complex, multi-gene nature of AD pathophysiology. Our results demonstrate that this approach not only significantly outperforms state-of-the-art models but also derives its strength from synergistically integrating diverse biological information, with curated pathways (C2) and ontology gene sets (C5) forming its predictive core.

The biological relevance of HyperAD’s predictions is strongly supported by multiple, independent lines of validation. Our top-ranked candidate genes are significantly enriched in hallmark AD biological processes, such as synaptic regulation and amyloid-beta clearance, and are densely connected to known AD genes in biological networks. Most compellingly, by leveraging proteomics data from the ROSMAP cohort, we show a direct link between the expression levels of our predicted genes and the clinical progression of cognitive decline. This convergence of computational and clinical evidence provides high confidence in our prioritized candidates as genuine players in AD.

While HyperAD marks a significant advance, we acknowledge its limitations. The model’s performance is dependent on the quality of existing gene set databases, and like all computational models, it predicts association, not causation. Therefore, the most critical next step is the experimental validation of our novel high-confidence candidates to confirm their functional roles. Furthermore, the HyperAD framework is inherently flexible and can be readily applied to other complex diseases, offering a powerful new tool for computational genomics.

In conclusion, HyperAD provides a more powerful and biologically intuitive approach to disease gene discovery. By shifting the paradigm from pairwise relationships to higher-order functional contexts, it delivers a robust and validated list of novel AD risk genes. These findings offer promising new avenues for research, with the potential to accelerate the development of future diagnostics and targeted therapies for Alzheimer’s disease.

## Data Availability

The original contributions presented in the study are included in the article/Supplementary Material, further inquiries can be directed to the corresponding author.
