# Supplementary material for: A hypergraph neural network for prioritizing Alzheimer’s disease risk genes
Source: Front Genet. 2025 Sep 19;16:1668200. doi: 10.3389/fgene.2025.1668200 (PMC12490983; doi:10.3389/fgene.2025.1668200)

***Supplementary Material*** for

**A Hypergraph Neural Network for Prioritizing Alzheimer's Disease Risk Genes**

**Supplementary Table**: The decile enrichment test results of 41 AD-associated biological processes

| **GO Accession** | **GO biological process name** | **FDR** |
| --- | --- | --- |
| GO:0050808 | synapse organization | 3.16E-57 |
| GO:0050890 | cognition | 7.72E-49 |
| GO:0007611 | learning or memory | 3.43E-47 |
| GO:0000302 | response to reactive oxygen species | 1.87E-46 |
| GO:0150076 | neuroinflammatory response | 1.45E-43 |
| GO:0050806 | synaptic transmission | 2.95E-41 |
| GO:0060078 | postsynaptic membrane potential | 1.09E-40 |
| GO:0042987 | APP catabolic process | 3.79E-40 |
| GO:0048167 | regulation of synaptic plasticity | 8.27E-37 |
| GO:0060079 | excitatory postsynaptic potential | 1.35E-36 |
| GO:2000377 | regulation of ROS metabolic process | 3.34E-36 |
| GO:0042982 | APP metabolic process | 2.59E-35 |
| GO:0099173 | postsynapse organization | 1.30E-33 |
| GO:1902991 | regulation of APP catabolic process | 1.01E-32 |
| GO:0034205 | amyloid-beta formation | 1.01E-32 |
| GO:1904645 | response to amyloid-beta | 5.09E-32 |
| GO:0099565 | chemical synaptic transmission | 2.73E-31 |
| GO:2000379 | positive regulation of ROS metabolic process | 4.21E-28 |
| GO:1902003 | regulation of amyloid-beta formation | 1.01E-26 |
| GO:1904646 | cellular response to amyloid-beta | 2.26E-26 |
| GO:0150077 | regulation of neuroinflammatory response | 2.99E-24 |
| GO:0097242 | amyloid-beta clearance | 3.96E-20 |
| GO:1902993 | positive regulation of APP catabolic process | 1.01E-19 |
| GO:1902004 | positive regulation of amyloid-beta formation | 1.01E-15 |
| GO:0050805 | negagtive regulation synaptic transmission | 1.25E-15 |
| GO:1900271 | long-term synaptic potentiation | 2.73E-14 |
| GO:1902992 | negative regulation of APP catabolic | 1.01E-13 |
| GO:2000463 | positive regulation excitatory postsynaptic potential | 1.72E-12 |
| GO:1902430 | negative regulation of amyloid-beta formation | 1.01E-11 |
| GO:1903426 | regulation of ROS biosynthetic process | 2.91E-11 |
| GO:1900221 | regulation of amyloid-beta clearance | 6.84E-08 |
| GO:1902959 | regulation of aspartic-type endopeptidase in APP cat | 1.01E-07 |
| GO:1903428 | positive regulation of ROS biosynthetic process | 4.52E-07 |
| GO:1990000 | amyloid fibril formation | 8.49E-07 |
| GO:0050435 | amyloid-beta metabolic process | 1.25E-06 |
| GO:0150094 | amyloid-beta clearance by catabolic | 1.00E-05 |
| GO:0007271 | synaptic transmission, cholinergic | 2.26E-05 |
| GO:1900223 | positive regulation of amyloid-beta clearance | 4.60E-04 |
| GO:0051124 | synaptic growth at neuromuscular junction | 8.56E-03 |
| GO:1905906 | regulation of amyloid fibril formation | 3.70E-03 |
| GO:1905907 | negative regulation of amyloid fibril formation | 2.80E-02 |

**Supplementary Figure**: Hyperparameter Sensitivity Analysis of HyperAD


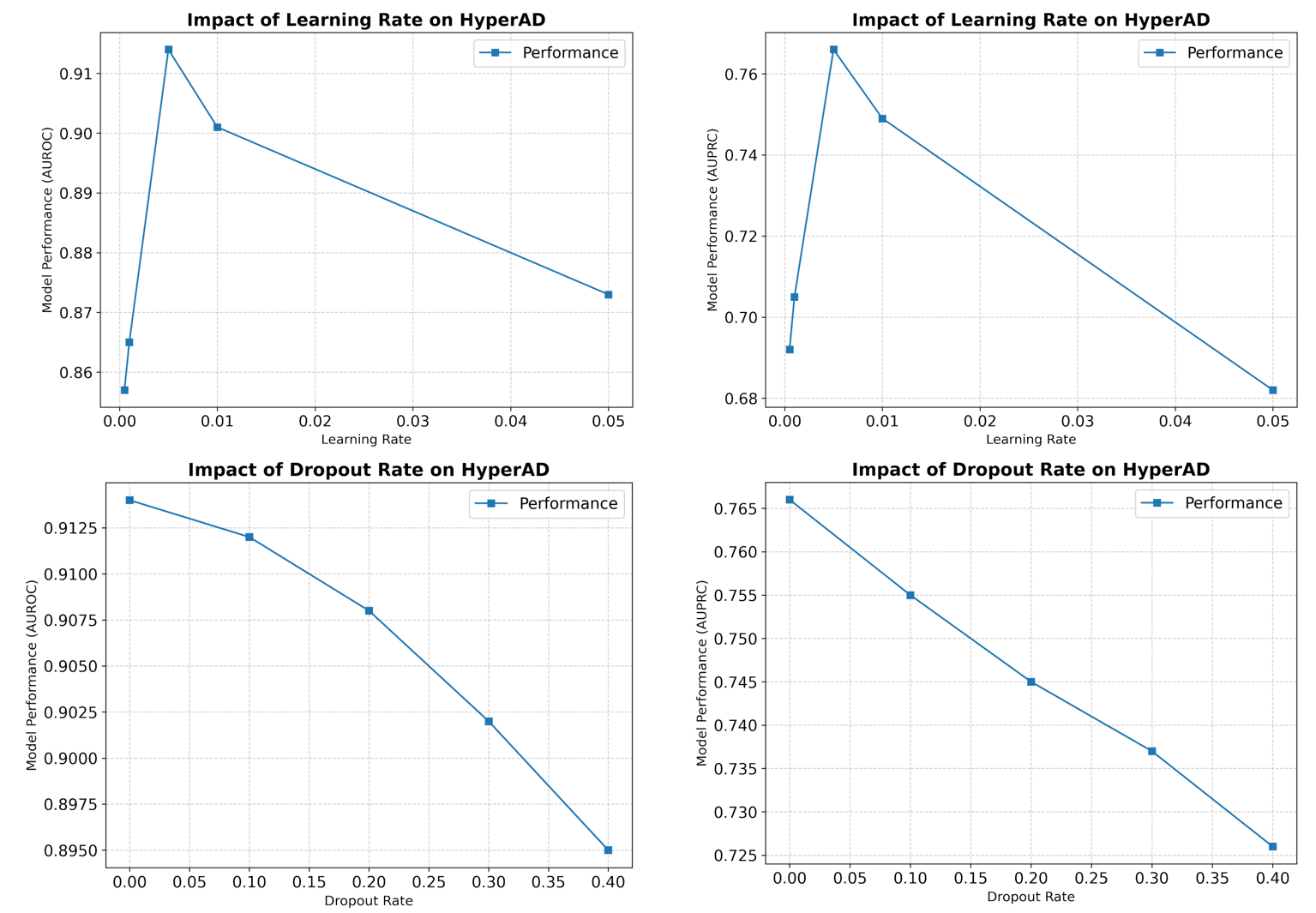

Supplement: Supplementary file 1 [file DataSheet1.docx]
